# Isolation of nanomolar scFvs of non-human primate origin, cross-neutralizing botulinum neurotoxins A_1_ and A_2_ by targeting their heavy chain

**DOI:** 10.1186/s12896-015-0206-0

**Published:** 2015-09-17

**Authors:** Arnaud Avril, Sebastian Miethe, Michel R. Popoff, Christelle Mazuet, Siham Chahboun, Christine Rasetti-Escargueil, Dorothea Sesardic, Philippe Thullier, Michael Hust, Thibaut Pelat

**Affiliations:** Département des Maladies Infectieuses, Institut de Recherche Biomédicale des Armées, Unité Interaction Hôte-Pathogène, 1 Place du Général Valérie André, BP73, 91220 Brétigny-sur-Orge, CEDEX France; Technische Universität Braunschweig, Institut für Biochemie, Biotechnologie und Bioinformatik, Abteilung Biotechnologie, Spielmannstr. 7, 38106 Braunschweig, Germany; Institut Pasteur, Centre National de Référence des bactéries anaérobies et du botulisme, 75724 Paris, France; Division of Bacteriology, National Institute for Biological Standards and Control (NIBSC), a centre of Medicines and Healthcare products Regulatory Agency, Blanche Lane, South Mimms, Potters Bar, Hertfordshire, EN6 3QG UK; BIOTEM, Parc d’activité Bièvre Dauphine 885, rue Alphonse Gourju, 38140 Apprieu, France

**Keywords:** Botulinum neurotoxin, Recombinant antibodies, scFv, Neutralizing antibodies, Non-human primates, Clostridium botulinum, AntiBotABE, Macaques, Biological warfare agents

## Abstract

**Background:**

Botulism is a naturally occurring disease, mainly caused by the ingestion of food contaminated by the botulinum neurotoxins (BoNTs). Botulinum neurotoxins are the most lethal. They are classified among the six major biological warfare agents by the Centers for Disease Control. BoNTs act on the cholinergic motoneurons, where they cleave proteins implicated in acetylcholine vesicle exocytosis. This exocytosis inhibition induces a flaccid paralysis progressively affecting all the muscles and generally engendering a respiratory distress. BoNTs are also utilized in medicine, mainly for the treatment of neuromuscular disorders, preventing large scale vaccination. Botulism specific treatment requires injections of antitoxins, usually of equine origin and thus poorly tolerated. Therefore, development of human or human-like neutralizing antibodies is of a major interest, and it is the subject of the European framework project called “AntiBotABE”.

**Results:**

In this study, starting from a macaque immunized with the recombinant heavy chain of BoNT/A_1_ (BoNT/A_1_-HC), an immune antibody phage-display library was generated and antibody fragments (single chain Fragment variable) with nanomolar affinity were isolated and further characterized. The neutralization capacities of these scFvs were analyzed in the mouse phrenic nerve-hemidiaphragm assay.

**Conclusions:**

After a three-round panning, 24 antibody fragments with affinity better than 10 nM were isolated. Three of them neutralized BoNT/A_1_ efficiently and two cross-neutralized BoNT/A_1_ and BoNT/A_2_ subtypes in the mouse phrenic nerve-hemidiaphragm assay. These are the first monoclonal human-like antibodies cross-neutralizing both BoNT/A_1_ and BoNT/A_2_. The antibody A1HC38 was selected for further development, and could be clinically developed for the prophylaxis and treatment of botulism.

**Electronic supplementary material:**

The online version of this article (doi:10.1186/s12896-015-0206-0) contains supplementary material, which is available to authorized users.

## Background

Botulism is a rare life-threatening disease caused by botulinum neurotoxins (BoNT), secreted by the spore-forming bacterium, *Clostridium botulinum*. Seven BoNTs serotypes (A to G) have been described but serotypes A, B and E are responsible for the majority of natural human intoxications [1]. Botulinum neurotoxin A (BoNT/A) is the most toxic substance known, with a human 50 % lethal dose (LD_50_) estimated at 1 ng.kg^−1^ (intravenous and subcutaneous routes), 10 ng.kg^−1^ (pulmonary route) or 1 μg.mL^−1^ (oral route), leading the Centers for Disease Control and Prevention (CDC) to classify BoNTs among the six major biological warfare agents (CDC “category A” agents) [2–4]. BoNTs are type A-B heteromeric molecules composed of a 100 kDa heavy chain (HC) and a 50 kDa light chain (LC). The heavy chain is successively implicated in the toxin binding at the surface of the motoneurons, the internalization by dual-receptor-mediated endocytosis and the translocation of the light chain into the cytosol. The light chain is a zinc endopeptidase which cleaves proteins of the SNARE (soluble N-ethylmaleimide-sensitive factor attachment protein receptor) complex, which is implicated in the exocytosis of the acetylcholine [5]. The SNAP25 (synaptosomal-associated protein of 25 kDa) protein is cleaved by BoNT/A-LC. This inhibition induces a flaccid muscular paralysis, affecting progressively all the muscles and generally leading to a respiratory distress and eventually death in absence of treatment. Vaccines against botulism have been developed but vaccination is rarely used as its effectiveness has not been fully evaluated and has demonstrated adverse events [6]. Thus, vaccination is only used for individuals with a high risk of exposure, such as health care providers, researchers, first responders, and military personnel [7]. Moreover, BoNTs have been introduced as a safe and effective treatment for a wide range of disorders associated with involuntary muscle contractions and spasm disorders, and these ever-increasing medical indications prevent large scale vaccination against botulism [8]. Currently, there is no licensed small inhibitor available in the pharmacopeia. Thus, the botulism specific treatment is based on the injection of anti-toxin antibodies, which should be administered as soon as possible after the clinical diagnosis to reduce the mortality rate, complemented by several months of hospitalization in an intensive care unit with mechanical ventilation [2, 9]. Infant botulism cases are treated by injections of a human-derived botulinum immunoglobulin preparation (BabyBIG®), which is well tolerated, but available in very limited quantity and is expensive [10]. Adult botulism is treated by injections of a bivalent (Sanofi-Pasteur Limited–Institut Butantan) or heptavalent (Cangene Corporation) horse-derived antitoxins, available in larger quantity. Nevertheless, because of their animal origin, these antitoxins may be poorly tolerated and could induce serious adverse effects like serum sickness or anaphylactic shock [11, 12].

The development of human or human-like antibodies for passive vaccination is advised as an alternative. In a former study, recombinant antibody fragments were isolated from an immune and a non-immune human library against botulinum toxins [13]. The immune library was built starting from a volunteer immunized with botulinum toxoids A to E, and yielded the single chain Fragment variables (scFvs) with best affinities and neutralization properties compared to those obtained from the naïve library [for review of the scFv format see: [14]. It was previously shown that the combination of antibodies targeting the heavy and the light chains of BoNT/A neutralize synergistically this toxin [15]. However, it is not always possible to immunize human volunteers with the antigen of interest, particularly in the case of biological warfare agents. For the construction of immune libraries, human volunteers may be substituted by non-human primates. in particular, it was demonstrated that macaque antibodies are close to their human counterparts, which might suggest a good tolerance in therapy [16, 17]. Non-human primate immunizations allow generation of immune gene-libraries focused on the antigen, from which high-affinity antibodies can be isolated. This strategy was previously utilized for the isolation of antibodies neutralizing biological warfare agents [18–21]. Within the context of the AntiBotABE project (http://www.antibotabe.com), this strategy was previously used successfully to isolate neutralizing antibodies directed against the light chain of BoNT/A [22–24].

In this work, two antibodies cross-reacting with and cross-neutralizing both botulinum neurotoxin sub-types A_1_ and A_2_ (BoNT/A_1_ and BoNT/A2), were generated.

## Results

### Animal immunization and phage-displayed library construction

**Fig. 1:**
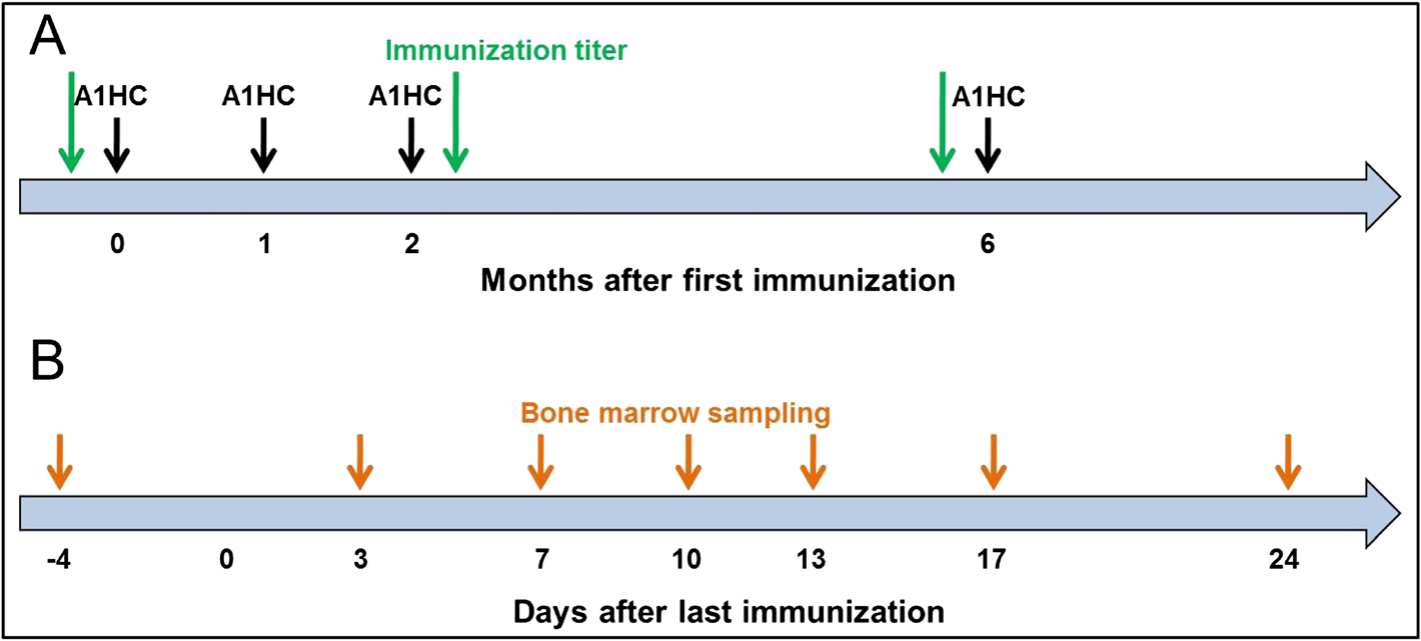
Scheme of macaque hyper-immunization and bone marrow sampling. **a** Scheme of macaque hyper-immunization. Four injections of the immunogen were performed, and sera were sampled to estimate the immunization titer. **b** Scheme of the bone marrow sampling, from 4 days before the first immunization up to 24 days after the last

A macaque (*Macaca fascicularis*) was hyper-immunized with four injections (referred to as immunizations) of a recombinant protein representing the binding domain (region 872–1296) of BoNT/A_1_ heavy chain (Fig. 1a). Thirteen day after the third immunization, a titer of 1/800.000, 1/1.310.000 and 1/40.000 was observed in ELISA against BoNT/A_1_-HC (the immunogen), BoNT/A_1_ (holotoxin) and BoNT/A_2_ (toxin as a complex form), respectively, confirming that sufficiently high immune response for the generation of a phage-display immune-library had been reached. Four months after the third immunization, the decrease of the macaque immune response was checked; the macaque bone marrow was drawn (Fig. 1a), and as expected, no significant PCR amplification of the DNA coding for the Fd fragment of the gamma heavy chain (Fd γ) and for the variable region of the kappa light chain (VLκ) of the antibodies was observed. This absence of amplification is due to the long period of time separating the third immunization and this sampling (see Additional file 1), and confirmed that the resting period was sufficiently long to decrease the immune response of the macaque. The corresponding profile of amplification was used as a negative control to observe the increase of the immune response after the fourth (and last) immunization. After the last immunization with BoNT/A_1_-HC, the bone marrow was iteratively sampled during 24 days (Fig. 1b). Using the same specific primers set as before the final immunization, the amplification of the PCR products increased and reached optimum 10 days after the last immunization, where all primer pairs were strongly amplified compared to the other days (see Additional file 1). Because the macaque was maintained in an animal house preventing environmental contamination, its humoral immunity was not stimulated with antigens other than injected-BoNT/A_1_-HC. As a consequence, just before the fourth immunization, no PCR amplification of the genes coding for the antibody variable regions was observed. The PCR products amplified at the 10th day were thus regarded as specifically encoding variable regions of antibodies directed against BoNT/A_1_-HC. Fd fragments of the γ heavy chain and κ light chain products of amplification were separately pooled and inserted into pGEM®-T vector to construct, two sub-libraries presenting diversities of 3.3 × 10^4^ clones and 1 × 10^5^ clones, respectively. These two sub-libraries were combined by two consecutive cloning steps into the pHAL14 vector [25–27] to generate the immune-library. First, VL gene fragments were cloned, using *Mlu*I and *Not*I, and then VH fragments, using *Nco*I and *Hind*III. The final scFv library consisted of 6.9 × 10^7^ independent clones with a full-size insert rate of 79 % and was finally was packaged in M13K07 phage.

### Panning of the library for the isolation of scFv directed against BoNT/A_1_

The phage-displayed scFvs library was subjected to 3 rounds of panning against BoNT/A_1_ holotoxin with the number of washes doubling after each round (5, 10 then 20 washes). After each round, only the phage having interacted with BoNT/A_1_ were eluted. Between the first and the second round of panning the number of eluted phage decreased from 115,000 (round 1) to 10,000 (round 2), underlining the selection of the small proportion of reactive phage among the whole immune-library (see Additional file 2). Between the second and the third round of panning, the number of eluted phage increased from 10,000 to 600,000 (round 3), which corresponds to the strong amplification and selection of reactive phage. The reactivity of the phage eluted after each round against BoNT/A_1_ was assessed in phage-ELISA and, the signal increased 9-fold between rounds 1 and 3, confirming the enrichment in scFvs highly reactive against the target (see Additional file 2). No reactivity was observed against KLH or BoNT/B_1_, indicating that eluted phage were highly specific for BoNT/A_1_ and that no cross-serotype reactivity was observed. The input titer of phage used for each round of panning was determined each day, and always comprised between 10^10^ and 10^12^ phage particles (data not shown).

### Isolation of anti-BoNT/A_1_ scFvs and affinity determination

One hundred clones eluted after the last round of panning were randomly chosen and their DNA was extracted and sequenced. The DNA sequences were translated into peptidic sequences with ExPASy Transalte tool. The sequences with an early stop codon or with an incorrect size were considered as recombined. The non-recombined sequences were aligned with ExPASy Decrease Redundancy tool, to identify the identical sequences (referred to as “redundant” sequences). Sixty-four sequences corresponding to non-recombined and non-redundant scFv sequences were identified. Fifty two of them (81.25 % of the non-recombined and non-redundant sequences) were found in a single copy whereas the other 12 sequences (18.75 %) were found in at least two copies, after the analysis of sequences redundancy, which gave an overview of the high diversity of the antibody fragments isolated after the phage-display screening. The over-represented sequences identified after the sequencing could correspond to clones preferentially amplified during the panning, because of their better interaction with the antigen. After transformation of the DNA in *E. coli* HB2151, the 64 different sequences were expressed as soluble scFv and purified. Their affinities for BoNT/A_1_ were measured by surface plasmon resonance and ranged from 1.3 nM (A1HC49) to 50 nM. The affinity of the scFv A1HC38 was measured at 1.9 nM with K_on_ = 6.34 × 10^4^ M^−1^.S^−1^ and K_off_ = 1.2 × 10^−4^ S^−1^ (see Additional file 3]). The twenty-four scFvs (37.5 % of all non-redundant and non-recombined scFvs) presenting affinities better than 10 nM (Table 1) were selected to test their neutralization capacities.

**Table 1:** List of the 24 scFvs with affinities better than 10 nM

Clone name	Affinity (nM)	Clone name	Affinity (nM)
A1HC49	1.3	A1HC68	3.3
A1HC7	1.4	A1HC81	3.3
A1HC31	1.5	A1HC65	4
A1HC80	1.6	**A1HC17**	**4.79**
**A1HC38**	**1.9**	A1HC39	5
A1HC6	2.2	A1HC43	5
A1HC74	2.3	A1HC58	5
A1HC32	2.6	A1HC64	5
A1HC47	2.6	A1HC34	5.1
A1HC67	3.1	A1HC62	5.1
A1HC26	3.2	**A1HC45**	**5.25**
A1HC33	3.3	A1HC3	6.3

Affinities were determined by surface plasmon resonance. The scFv were run in flux in a sensor chips coated with BoNT/A_1_. The scFvs in bold were of particular interest for this study

### Computational analyse

After the isolation and the sequencing of the scFvs, a computational analysis using IMGT/V-QUEST tool was performed to retrieve the human germline sequences closer to the sequence of the 24 selected scFvs. As shown in Table 2, the use of three IGHV family genes (families IGHV-1, -3 and -5) was observed: 15 occurrences of IGHV3-49*03 plus one occurrence of IGHV3-21*04 and one occurrence of IGHV3-71*01, 3 occurrences of IGHV1-69*04, and 2 occurrences of IGHV5-51*01 and IGHV5-a*04. These VH were combined to four different IGHJ family genes (families IGHJ-2, -4, -5 and -6). Regarding light chains, the use of two different IGKV genes (1 and 3) was observed: IGKV1-39*01 and IGKV1 -16*01 (5 occurrences each), IGKV1-17*01 (4 occurrences), IGKV1-27*01, IGKV1-13*02 and IGKV1-9*01 (2 occurrences each) and finally one occurrence of IGKV1D-13*01, plus three single occurrence of IGKV3 (IGKV3-7*02, IGKV3-11*01 and IGKV3-20*01). These IGKV genes are also combined to different IGKJ family genes (families IGKJ-1, -2, -3 or -4). All these rearrangements result in a high diversity of the selected scFv sequences.

**Table 2:** Human germline genes closer to the genes coding for the 24 scFvs with affinities better than 10 nM

	Heavy chain			Light chain	
Clone name	V	D	J	V	J
A1HC3	IGHV3-49*03	IGHD4-23*01	IGHJ4*02	IGKV1D-13*01	IGKJ3*01
A1HC6	IGHV3-21*04	IGHD4-17*01	IGHJ5*02	IGKV3-7*02	IGKJ3*01
A1HC7	IGHV3-49*03	IGHD4-23*01	IGHJ4*02	IGKV1-39*01	IGKJ4*01
**A1HC17**	IGHV5-51*01	IGHD6-19*01	IGHJ5*02	IGKV3-11*01	IGKJ3*01
A1HC26	IGHV3-49*03	IGHD4-23*01	IGHJ4*02	IGKV1-17*01	IGKJ4*01
A1HC31	IGHV3-49*03	IGHD4-23*01	IGHJ4*02	IGKV1-27*01	IGKJ2*03
A1HC32	IGHV5-a*04	IGHD1-26*01	IGHJ5*02	IGKV1-13*02	IGKJ1*01
A1HC33	IGHV3-49*03	IGHD4-23*01	IGHJ4*02	IGKV1-13*02	IGKJ4*01
A1HC34	IGHV3-49*03	IGHD4-23*01	IGHJ4*02	IGKV1-16*01	IGKJ4*01
**A1HC38**	IGHV5-a*04	IGHD1-26*01	IGHJ5*02	IGKV1-39*01	IGKJ3*01
A1HC39	IGHV3-49*03	IGHD4-23*01	IGHJ4*02	IGKV1-16*01	IGKJ4*01
A1HC43	IGHV1-69*04	IGHD3-22*01	IGHJ6*02	IGKV1-27*01	IGKJ3*01
**A1HC45**	IGHV3-49*03	IGHD4-23*01	IGHJ4*02	IGKV1-16*01	IGKJ4*01
A1HC47	IGHV3-49*03	IGHD4-23*01	IGHJ4*02	IGKV1-39*01	IGKJ4*01
A1HC49	IGHV3-49*03	IGHD4-23*01	IGHJ4*02	IGKV1-16*01	IGKJ2*01
A1HC58	IGHV3-49*03	IGHD4-23*01	IGHJ4*02	IGKV3-20*01	IGKJ3*01
A1HC62	IGHV5-51*01	IGHD5-12*01	IGHJ2*01	IGKV1-17*01	IGKJ4*01
A1HC64	IGHV3-49*03	IGHD4-23*01	IGHJ4*02	IGKV1-9*01	IGKJ2*03
A1HC65	IGHV3-71*01	IGHD4-23*01	IGHJ4*02	IGKV1-9*01	IGKJ1*01
A1HC67	IGHV1-69*04	IGHD2-15*01	IGHJ6*02	IGKV1-17*01	IGKJ2*01
A1HC68	IGHV3-49*03	IGHD4-23*01	IGHJ4*02	IGKV1-39*01	IGKJ4*01
A1HC74	IGHV1-69*04	IGHD2-15*01	IGHJ6*02	IGKV1-17*01	IGKJ2*03
A1HC80	IGHV3-49*03	IGHD4-23*01	IGHJ4*02	IGKV1-39*01	IGKJ3*01
A1HC81	IGHV3-49*03	IGHD4-23*01	IGHJ4*02	IGKV1-16*01	IGKJ4*01

Human germline genes closer to the genes coding for the 24 best scFvs were retrieved using IMGT/V-QUEST. The scFvs in bold were of particular interest for this study

Germinality index (GI) represents the percentage of identity at the amino acids level between the framework regions of a scFv and the framework regions encoded by the corresponding human germline V and J sequences [18, 28]. The GI of the 24 best scFvs are presented in Table 3; values ranged between 81.13 % and 87.72 %, underlining their high identity level with human sequences and thus their potential low immunogenicity. The G-score is another parameter that could indirectly predict the tolerance of the scFv, but it is based on comparison with the expressed genes and not with germline genes [29]. The G-scores of the 24 selected scFv were also determined and ranged between −1.01 and −2.37 (see additional file 4). Even if the G-score of the light chains of A1HC34, A1HC45 and A1HC65 were positives, all mean G-score were negatives.

**Table 3:** Germinality index of the 24 scFvs with affinities better than 10 nM

	Germinality Index (GI)		
Clone name	Heavy chain	Light chain	Mean GI
A1HC58	84.44	91.01	87.72
A1HC81	82.41	92.13	87.27
A1HC65	78.01	95.5	86.75
A1HC33	82.41	91.01	86.71
A1HC62	83.51	89.77	86.64
A1HC32	83.33	89.53	86.43
A1HC26	80.21	92.13	86.17
**A1HC45**	**80.21**	**92.13**	**86.17**
A1HC3	79.12	92.85	85.98
A1HC67	85.71	86.04	85.87
**A1HC17**	**80.21**	**89.88**	**85.04**
A1HC74	84.61	85.21	84.91
**A1HC38**	**82.22**	**86.51**	**84.36**
A1HC64	78.02	89.88	83.95
A1HC68	78.02	89.88	83.95
A1HC49	80.21	87.64	83.92
A1HC31	78.02	89.77	83.89
A1HC34	79.12	88.37	83.74
A1HC43	83.51	83.52	83.52
A1HC7	78.02	88.76	83.39
A1HC39	79.12	86.51	82.81
A1HC80	76.92	86.36	81.64
A1HC6	90.69	71.91	81.3
A1HC47	79.12	83.14	81.13

The Germinality Index (GI) of the 24 scFvs with the best affinities was calculated for each sequence (VH, VL, mean GI) using IMGT/DomainGapAlign tool. The scFvs were ranked according to their predictive tolerance. The scFvs in bold were of particular interest for this study

### scFvs neutralization capacities

**Fig. 2:**
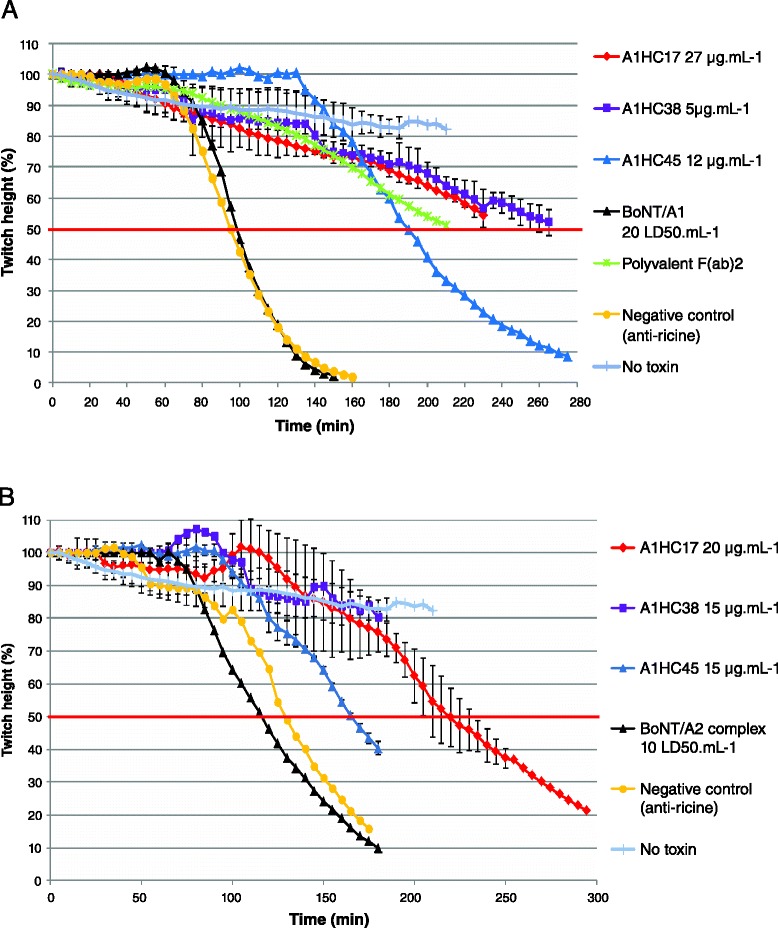
Neutralization of BoNT/A_1_ and BoNT/A_2_ by high concentration of scFvs in the mouse phrenic nerve-hemidiaphragm assay. **a** Neutralization of purified-BoNT/A_1_ holotoxin (20 LD_50_.ml^−1^) with A1HC17, A1HC38 or A1HC45 at a single high concentration. **b** Neutralization of complexed-BoNT/A_2_ (10 LD_50_.ml^−1^) with A1HC17, A1HC38 or A1HC45 at a single high concentration. BoNT/A_1_ and BoNT/A_2_ were premixed with 27, 20, 15, 12 or 5 μg.mL^−1^ of A1HC17, A1HC38 or A1HC45 or with the commercial polyvalent F(ab’)_2_ antitoxin (activity of 20 mIU.mL^−1^ against BoNT/A). The toxins alone were used as controls to determine the time required to observed a 50 % decrease in the twitch height. No significant neutralization was observed with an irrelevant scFv directed against the ricin toxin (referred as “negative control”), tested at a single concentration of 9 μg.mL^−1^ in all experiments. The experiments were run until a decrease of at least 50 % in the twitch height was observed, until the phrenic nerve hemidiaphragm preparation was no longer viable or until no more twitch was detected. Control tissues, not exposed to the toxin were included to demonstrate stability of recordings (referred as “No toxin”)

**Fig. 3:**
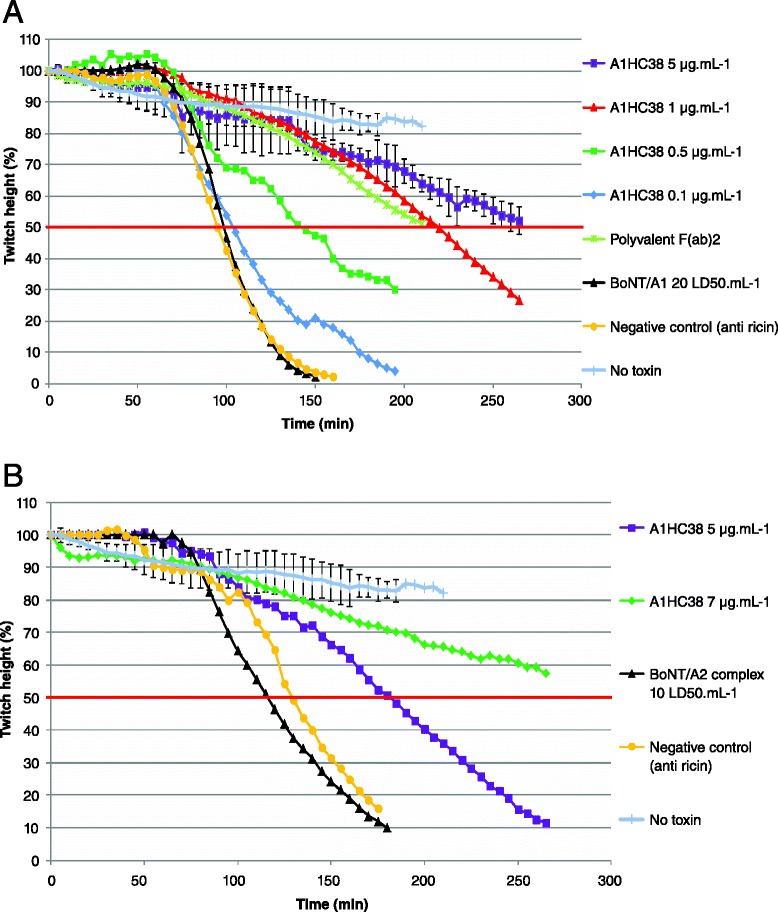
Neutralization of BoNT/A_1_ and BoNT/A_2_ by the scFv A1HC38 in the mouse phrenic nerve-hemidiaphragm assay. Neutralization of BoNT/A_1_ and BoNT/A_2_ by decreasing concentrations of the scFv A1HC38. **a** Neutralization of BoNT/A_1_ holotoxin (20 LD_50_.ml^−1^) induced by scFv A1HC38 at decreasing concentrations. **b** Neutralization of BoNT/A_2_ as a form of complexes (10 LD_50_.ml^−1^) induced by the scFv A1HC38 at decreasing concentrations. The toxins were premixed with 7, 5, 1, 0.5 or 0.1 μg.mL^−1^ of A1HC38 or with 20 mIU.mL^−1^ (anti BoNT/A antibodies) of the commercial polyvalent F(ab’)_2_ antitoxin. The toxins alone were used as controls to determine the time required to observed a 50 % decrease in the twitch height. No significant neutralization was observed with an irrelevant scFv directed against the ricin toxin (referred as “negative control”), tested at a single concentration of 9 μg.mL^−1^ in all experiments. The experiments were run until a decrease of at least 50 % in the twitch height was observed, until the phrenic nerve hemidiaphragm preparation was no longer viable or until no more twitch was detected. Control tissues, not exposed to the toxin were included to demonstrate stability of recordings (referred as “No toxin”)

All scFvs with affinities better than 10 nM were characterized in the *ex vivo* mouse phrenic nerve-hemidiaphragm assay to identify those neutralizing BoNT/A_1_ holotoxin and those cross-neutralizing BoNT/A_2_ as a complex (Figs. 2 and 3). First, the 24 selected scFvs were screened at the highest possible concentration (scFv volume less than 10 % of tissue bath volume) against BoNT/A_1_ to identify the scFvs presenting some neutralization capacities (Fig. 2a). Then, only the scFv significantly neutralizing BoNT/A_1_ were characterized at higher concentration against BoNT/A_2_ (Fig. 2b), to identify the cross-neutralizing scFvs. Finally, the best scFv cross-neutralizing BoNT/A_1_ and BoNT/A_2_ was more precisely characterized against BoNT/A_1_ and BoNT/A_2_, using decreasing concentrations of the scFv (Fig. 3). Standard deviations are shows only for the concentrations of particular interest. In both figure, 43RCA, a scFv directed against the ricin toxin, was used as a negative control of neutralization [21]. In the presence of this irrelevant scFv, the 50 % decrease in muscle concentration was always delayed by less than 10 min, which was considered as non-significant (Figs. 2 and 3). In absence of toxin, only a slow decrease of the muscle contraction is observed, due to the progressive, but weak, degradation of the muscle preparation (Figs. 2 and 3).

As shown in Fig. 3a, in the presence of BoNT/A_1_ holotoxin alone (purified toxin, 20 LD_50_.mL^−1^), the time to reach a 50 % paralysis was 97 min. The F(ab’)_2_ antitoxin (Botulismus–Antitoxin Berhring, Novartis) was used as a positive control for neutralization. The 50 % paralysis time with BoNT/A_1_ was delayed by 115 min in the presence of 20 mIU.mL^−1^ of this positive control (Fig. 2a). Among the 24 scFvs tested, 3 efficiently neutralized BoNT/A_1_ (20 LD_50_.mL^−1^) at the concentration of 27 μg.mL^−1^ (A1HC17), 5 μg.mL^−1^ (A1HC38) or 12 μg.mL^−1^ (A1HC45). At the concentrations tested these scFvs delayed the 50 % paralysis time by more than 145 min (145 %), by 190 min (190 %) and by 95 min (95 %), respectively. The three scFvs neutralizing BoNT/A_1_ were then tested at higher concentration against BoNT/A_2_ in the complex form. As shown in Fig. 2b, in the presence of complex BoNT/A_2_ alone (10 LD_50_.ml^−1^), the time to reach a 50 % paralysis was 118 min. As shown in Fig. 2b, at concentrations of 20 μg.mL^−1^ (A1HC17) or 15 μg.mL^−1^ (A1HC38 and A1HC45), the 50 % paralysis time was delayed by 100 min (84 %, A1HC17), more than 220 min (188 %, obtained by extrapolation, A1HC38) and 50 min (42 %, A1HC45).

As the scFv A1HC38 was the most efficient cross-neutralizing scFv, it was selected for further characterization at lower concentrations against BoNT/A_1_ and BoNT/A_2_ (Fig. 3). As shown in Fig. 3a, at concentrations of 0.5 and 1 μg.mL^−1^, A1HC38 delayed the 50 % paralysis time induced by BoNT/A_1_ (20 LD_50_.mL^−1^) by 45 and 120 min, respectively (45 and 120 % delay in paralysis, respectively). As no significant neutralization was observed with 0.1 μg.mL^−1^ of A1HC38, the minimum neutralizing concentration was considered to be close to 0.5 μg.mL^−1^. As shown in Fig. 3b, at concentrations of 7 and 5 μg.mL^−1^, A1HC38 delayed the paralysis induced by BoNT/A_2_ by more than 227 min (more than 192 % delay in paralysis) and by 65 min (55 % delay in paralysis), respectively.

## Discussion

BoNT/A is the most toxic substance currently known and its neutralization is of a major concern for public health and biological defense [3]. For this purpose, recombinant antibodies represent a potent tool because they can be used for both therapy and prophylaxis and they have proven their efficacy for the neutralization of biological warfare agents such as ricin, anthrax or botulinum neurotoxins [18, 20, 21, 23]. Several studies have previously isolated antibodies neutralizing BoNT/A by targeting the light chain or the heavy chain [15, 22, 23, 30–34]. Nevertheless, only few of them are of human or human-like origin and they generally neutralized only one of the five subtypes currently described [22, 23, 30, 34–36]. It was demonstrated that the combination of several antibodies synergistically neutralized BoNT/A, particularly if one antibody is directed against the heavy chain of BoNT/A (BoNT/A-HC) and another against its light chain (BoNT/A-LC) [15, 31]. Recently, we have generated scFvs directed against the light chain of BoNT/A_1_ (BoNT/A_1_-LC) [22, 37]. These scFvs inhibited the catalytic activity of the BoNT/A_1_ subtype and neutralized this toxin in the *ex vivo* phrenic nerve assay. In this work, human-like scFvs neutralizing BoNT/A were generated. Antibodies isolated from immune libraries were generally more neutralizing than antibodies isolated from naïve libraries [15, 31]^.^ Because human subjects cannot be immunized with bio warfare agents, our strategy consisted in substituting them with the non-human primates for immunization. Non-human primate antibody sequences are close to human ones and thus would be useful for the isolation of well tolerated antibodies. We previously showed that macaque antibodies are significantly more similar to human antibody sequences compared to chimpanzee antibody sequences [16]. Thus, in the present study, an immune library was constructed starting from a macaque *(Macaca fasciculari*s) immunized with a non-toxic recombinant fragment corresponding to the C-terminus of BoNT/A_1_-HC. This immune library was panned against BoNT/A_1_ holotoxin. The best scFvs with affinities below 10 nM were evaluated for their neutralization capacities. To identify cross-neutralizing scFvs, their capacity to neutralize both pure BoNT/A_1_ holotoxin and complex BoNT/A_2_ was successively evaluated in the *ex vivo* mouse phrenic nerve-hemidiaphragm assay [38]. This test closely mimics the *in viv*o respiratory paralysis and the results obtained for neutralization of polyclonal antibodies closely relate to the results obtained in the mice lethality assay. The BoNT/A_2_ toxin used for this test corresponded to toxin in the form of complexes and not of purified holotoxin. During the panning it was not possible to use complexed toxin because it would have led to the isolation of non-neutralizing antibodies directed against the others proteins (hemagglutinin and non-hemagglutinin proteins) constituting these complexes. In this *ex vivo* test, all scFvs which significantly delayed the 50 % reduction in muscle twitch were considered as neutralizing. Among the 24 scFvs tested, A1HC17, A1HC38 and A1HC45 neutralized efficiently BoNT/A_1_ holotoxin. These three scFvs were further characterized against complexed BoNT/A_2_. Two of them, A1HC17 and A1HC38, were identified as cross-neutralizing, and A1HC38 was identified as the most potent scFv. At a concentration of 5 μg.mL^−1^, A1HC38 delayed the paralysis induced by BoNT/A_1_ (20 LD_50_.mL^−1^) by 164 % and the paralysis induced by BoNT/A_2_ (10 LD_50_.mL^−1^) by 55 %. A non-linear dose-effect was observed. These results are promising as generally an efficient neutralization of BoNT/A requires the association of several neutralizing antibodies and because neutralization properties of BoNT/A_1_ by A1HC38 were similar or even better than the neutralization observed using the polyclonal F(ab’)_2_ antitoxin at activity of 20 mIU.mL^−1^ against BoNT/A which was included as a positive control [15, 31]. Antibodies targeting the heavy-chain could neutralize the toxin by different mechanisms, mainly by preventing toxin binding and its internalization into the motoneurons, but they could also indirectly inhibit the catalytic activity, by steric hindrance. The affinity of A1HC17 and A1HC38 were measured and were equal to 4.79 and 1.9 nM, respectively, and it is interesting to note that no strict correlation between high affinity and neutralization properties was observed; in fact the four scFvs with the highest affinities were not neutralizing.

The antibody-library was constructed starting from an immunized non-human primate. Even though close to their human counterpart, macaque IgG are not identical and could induce the formation of an anti-drug antibodies (ADA) responses by the patients treated with these molecules [29]. This ADA response could lead to decreasing the efficiency of the therapeutic antibody due to its quick elimination from the bloodstream. To predict the antibody tolerance, two different mathematical indexes were used: the Germinality Index (GI) [20, 28] and the G-score [29]. These parameters compare the variable region of a given antibody with the human germline V(D)J genes, and with expressed antibodies, respectively. The scFvs A1HC17 and A1HC38 had a mean GI equal to 85.04 % and 84.36 %, respectively, underlining a high proximity to human antibody framework sequences. For comparison, the mean GI values of 100 unpublished human scFv from naive antibodies libraries are 96.6 % for VH and 94.8 % for VL [23]. The mean G-score of A1HC17 and A1HC38 were −1.53 and −1.75, respectively, thus they were respectively “as human” as 6 and 4 %, of the human antibodies belonging from the same germline gene family present in the Kabat database. A sequence with a G-score equal to zero has the same identity level with human expressed antibodies than the average identity observed when human antibodies are compared between them. A sequence with a negative G-score present lower than average identity level and should be humanized to increase its tolerance. To decrease the immunogenicity, the variable domain of the scFvs should be germline-humanized. This strategy was previously utilized for the humanization of another antibody fragment (Fragment antigen binding, Fab) called 35PA_83_, whose germline humanization increased its GI from 87.6 to 97.8 % and its G-scores from 0.1945 to 0.265, predicting a better tolerance [39]. Because A1HC17 and A1HC38 cross-neutralized sub-types BoNT/A_1_ and BoNT/A_2_, they are good candidates for future clinical development, especially in combination with the anti-light chain antibody SEM120-IIIC1 [23].

## Conclusions

In the context of the European AntiBotABE project, an immune phage-display library was constructed starting from a macaque (*Macaca fascicularis*) immunized with a non-toxic fragment corresponding to the C-terminus of BoNT/A_1_ heavy-chain. The screening of this immune library led to the isolation of several neutralizing scFvs. The scFv A1HC38, with an affinity of 1.9 nM, and cross-neutralized efficiently for both BoNT/A_1_ and BoNT/A_2_ in the *ex vivo* phrenic nerve-hemidiaphragm assay was the most potent of the isolated scFv. Furthermore, scFv A1HC38 presented a Germinality Index of 84 % and a G-score of −1.75. To ensure excellent bioavailability and tolerance, the scFv could be germline-humanized and then reformatted as full-sized IgG and neutralizing potency assessed *in vivo*.

## Methods

### Ethical statement

The experiment in macaque was approved and performed in compliance with all relevant French ethical guidelines and laws, in particular (i) “Partie réglementaire du livre II du code rural (Titre I, chapitre IV, section 5, sous-section 3 : expérimentation sur l’animal)”, (ii) “ Décret 87–848 du 19/10/1987 relatif aux expériences pratiquées sur les animaux vertébrés modifié par le décret 2001/464 du 29/05/2001”, (iii) “ Arrêté du 29 octobre 1990 relatif aux conditions de l’expérimentation animale pour le ministère de la défense” and (iv) “instruction 844/DEF/DCSSA/AST/VET du 9 avril 1991 relative aux conditions de réalisation de l’expérimentation animale”.

Animal care procedures complied with the regulations detailed under the Animal Welfare Act and in the Guide for the Care and Use of Laboratory Animals [40, 41]. Animals were kept at a constant temperature (22 °C ± 2 °C) and relative humidity (50 %), with 12 h of artificial light per day. They were housed in individual cages (6 per room), each of which contained a perch. Animals were fed twice daily, once with dried food and once with fresh fruits and vegetables, and water was provided at the same time. Food intake and general behavior were observed by animal technicians during feeding times, and veterinary surgeons were available for consultation if necessary. Veterinary surgeons also performed systematic visits to each NHP-room twice weekly. The environmental enrichment program for the non-human primates consisted in games with animal care staff and access to approved toys. The well-being of the animals was monitored by the attending veterinary surgeon. Animals were anesthetized before the collection of blood or bone marrow by an intramuscular injection of 10 mg.kg^−1^ ketamine (Imalgene®, Merial). Analgesics were subsequently administered, through a single intramuscular injection of 5 mg.kg^−1^ flunixine (Finadyne®, Schering Plough) in the days after interventions if the animal technicians suspected that the animal was in pain, on the basis of their observations of animal behavior. None of the non-human primates were killed during this study.

### BoNT/A_1_ holotoxin and commercial equine polyclonal F(ab’)_2_

Purified BoNT/A_1_ hemagglutinin-free holotoxin was purchased from Metabiologics Inc (Madison, Wi, USA) at a concentration 2.3 × 10^8^ LD_50_.mg^−1^, (1 mg.mL^−1^ from the Hall strain, batch A062805–01) and diluted to 2 × 10^4^ LD_50_.mL^−1^ in gelatin (0.2 % w/v) phosphate (50 mM disodium hydrogen orthophosphate) buffer pH 6.5 (GPB), and stored frozen at −80 °C. The commercially-available equine polyclonal F(ab’)_2_ preparation against BoNT/A, B and E toxins (Novartis Vaccines), was used as positive control for neutralization..

### Animal immunization

Four subcutaneous immunizations with 90 μg of a protein representing the region 872–1296 of the Botulinum A_1_ Heavy Chain were performed in a male cynomolgus macaque (*Macaca fascicularis*) in Freund’s adjuvant (Sigma Aldrich). This recombinant BoNT/A_1_-HC fragment, corresponding to the binding domain of BoNT/A_1_, was prepared as previously described [42]. The three first immunizations were administered at one month intervals and the fourth immunization was administered four months after the third.

### ELISA assay

The macaque immune response was evaluated by ELISA performed as described previously, except that BoNT/A_1_ (Metabiologics Inc) was utilized instead of LF [20]. Briefly, a Maxisorb plate (Nunc) was coated overnight with 2 μg.mL^−1^ of BoNT/A_1_ in PBS. Then, after the coating, plates were saturated for 2 h at 37 °C with PBS-3 % BSA. Then animal serum was incubated for 2 h at 37 °C and the pre-immune serum was used as a negative control. The reactions were developed with the goat anti-human-IgG (Fc specific) HRP-conjugated antibody (Sigma Aldrich) diluted 1/1.000 in the saturation buffer and incubated for 1 h at 37 °C. Positive reaction for positive antibody binding were revealed by addition of TMB (3,3′,5,5′-tetramethylbenzidine, Sigma Aldrich) as the substrate, and read at 650 nm. The titer of the response was measured as the highest dilution of the immune serum giving a signal three times stronger than the negative control.

For phage-ELISA, an ELISA plate was coated overnight with 5 μg.mL^−1^ of BoNT/A_1_ in PBS. Plates were then saturated for 2 h at 37 °C in PBS-3 % milk. In parallel, eluted phage were diluted in PBS-1 % milk and incubated for 15 min at 37 °C before transfer into the wells and incubated for 2 h at 37 °C. The reactions were developed with the anti-M13 HRP conjugated mouse antibody (Amersham Biosciences), diluted 1/1.000 in PBS-1 % milk. Plates were revealed by addition of TMB and read at 650 nm.

### Construction of the anti-BoNT/A_1_-HC scFv phage display library

After the final immunization with the immunogen, the bone marrow of the macaque was sampled twice a week during the 3 weeks, with sample volume not exceeding 5 mL each. Total RNA was extracted using Tri Reagent (Molecular Research Center Inc). RNA coding Fd fragments of the γ chain and к light chains were retro-amplified with 9 and 7 primer sets, respectively, and the day where the amplification was considered as optimal was chosen to build the library [43]. The quality of the amplification was controlled in electrophoresis, with a 1 % TBE (sigma) / 0.8 % agarose-gel. The SmartLadder SF MW-1800-04 (Eurogentec) was used as reference during the electrophoresis. Fd fragment and к light chains PCR products of amplification were separately pooled and cloned in the pGEM®-T vector (Promega), according to the manufacturer’s instruction, to generate two distinct sub-libraries (one for the heavy and one for light chains). The two sub-libraries were separately electroporated (1800 Volts, 4 kΩ, 330 μF) in *Escherichia coli* SURE cells. The electroporated cells were resuspended in 1 mL of SOC media (Invitrogen) and incubated for 1 h at 37 °C. After incubation, 50, 5 and 0.5 μl of the cells were spread on petri dishes (composed of super broth media supplemented with 50 μg.mL^−1^ of carbenicillin) and incubated overnight at 37 °C. Th remaining cells were amplified overnight in 200 mL of SB media supplemented with 50μG.mL^−1^ of carbenicillin. The next day, the number of colonies (number of clones) on the petri dishes was counted and corresponded to the diversity of the two sub-libraries. The overnight cell cultures were separately centrifuged and the DNA was purified with the NucleoBond AX100 kit (Macherey Nagel), according to the manufacturer’s instruction. The DNA contained in the two sub-libraries, and encoding the VL (variable region of the light chain) or VH (variable region of the heavy chain) regions only, were then reamplified with five oligonucleotide primer sets introducing restriction sites allowing the cloning in pHAL14 [25–27]. The first step of the library construction consisted of the VL fragments cloning in pHAL14, and then the VH fragments were inserted into pHAL14 containing the VL repertoire. For this, the pHAL14 vector and the VL fragments were digested with MluI and NotI (New England Biolabs), the enzymes were inactivated, pHAL14 was dephosphorylated using calf intestinal phosphatase (MBI Fermentas), and the DNA was purified. VL PCR products (270 ng) were inserted into 1 μg of the dephosporylated pHAL14 preparation in four separate ligation reactions. DNA was precipitated from the reaction mixes with ethanol and sodium acetate, the pellet was washed twice with 70 % ethanol, and then four aliquots (25 μl) of XL1-Blue MRF’ (Stratagene) were used for electroporation. Plasmids (the VL library) were isolated using a Plasmid Midi Kit (QIAGEN). The VL library and the VH fragments were digested with *Nco*I and *Hind*III (New England Biolabs), and ligation and electroporation were then performed as described for VL. The library was packaged using M13K07 helper-phage (New England Biolabs). The titer of the phage-display immune-library was determined as described previously [44].

### Selection of recombinant antibodies against BoNT/A_1_ by phage display

Three wells of a Maxisorp plate (Nunc) were coated overnight at 4 °C with BoNT/A_1_ holotoxin at a concentration of 20 μg.mL^−1^ in PBS. Then, the wells were blocked with TBS-3 % BSA for 2 h at 37 °C, and washed with TBS-0.1 % Tween^®^ 20. The antibody phage display library was then incubated for additional 2 h at 37 °C. For the first round of panning, wells were washed five times (5 pipetting up and down per wash, with an interval of 5 min between each wash). After the last wash, the wells were rinsed with sterile TBS, and the phage were eluted with trypsin at 10 mg.mL^−1^, incubated in the wells at 37 °C for 30 min with shaking. Eluted phage were used to infect a 2 mL 0.5 OD *Escherichia coli* SURE strain (Stratagene) cultured at 37 °C in Super Broth media (Euromedex), supplemented with 10 μg.mL^−1^ of tetracycline. Culture volume was increased to 100 mL and supplemented with 10^12^ M13K07 helper phage (Life technology), tetracyclin (10 μg.mL^−1^), carbenicillin (50 μg.mL^−1^), kanamycin (25 μg.mL^−1^) and incubated overnight (30 °C). In parallel, 10^−4^ μl to 100 μl of this culture was spread on petri dishes (Super Broth media supplemented with carbenicillin) to determine the number of eluted clones. Overnight culture supernatant was precipitated for 30 min on ice with 0.4 % PEG 8000 (Sigma Aldrich) and 0.3 % NaCl, resuspended in TBS-1 % BSA-0.02 % NaN_3_, filtered (0.2 μm) and used for the next round of panning. The titer of precipitated phage, used for the following round of panning, was determined by infecting a 0.5 OD *Escherichia coli* SURE strain culture with the 10^−6^ to 10^−16^ μl of the phage production, during 15 min at 37 °C. The product of infection was spread on petri dishes (composed of super broth media supplemented with 50 μg.mL^−1^ of carbenicillin) and incubated overnight at 37 °C, before counting the number of bacterial colonies, which is referred as the phage titer. Two other rounds of panning, with 10 and 20 washes, were then performed using the same approach.

### scFv sequencing

One hundred clones were randomly handpicked from the third round of panning, and were cultured in 10 mL SB medium supplemented with carbenicillin (50 μg.mL^−1^). DNA was extracted using a QIAcube system (QIAgen, Courtaboeuf, France), according the manufacturer’s intructions. The DNA was digested with NotI and NcoI enzymes (New England Biolab), according to the manufacturer’s instructions and subjected to an electrophoresis in 0.5 % TBE and with a 0.8 % agarose–1 % TBE gel. The ladder AB-0387 (Thermo Scientific) was used as reference of size. The DNA inserts with an incorrect size were considered as recombined, and discarded. The non-recombined DNAs were sequenced by Beckman Coulter Genomics (Takeley, United-Kingdom). The DNA sequences provided by Beckman Coulter Genomics were converted into peptidic sequences with ExPASy Translate Tool (http://web.expasy.org). The sequences presenting an early stop codon or with an abnormal size were considered as recombined or unusable and were not selected. Then, the sequences were aligned with ExPASy Decrease Tedundancy tool to identify the identical sequences (http://web.expasy.org).

### scFv production and affinity determination

DNA encoding scFv was used to transform the non-suppressor *E. coli* strain HB2151 in order to express soluble scFv as previously described [20]. The expression of the scFvs was induced with IPTG (life technologies). The scFv were purified using nickel columns (QIAgen) and concentrations were estimated using capillary electrophoresis (Experion, Biorad).

The scFv affinities were measured by surface plasmon resonance using a BIAcore 3000 (GE-Healthcare), instrument. The toxin was coated at a maximum of 1100 resonance units (RU) on a CM5 chip (GE-Healthcare) *via* amine coupling, according to manufacturer’s instructions. A volume of 100 μl of at least five dilutions of the scFvs in HBS-EP buffer (GE-Healthcare), were tested. Generally sample dilution concentrations ranged from 2 μM to 0.1 nM. A 30 μL.min^−1^ flow rate was maintained during the run. After each scFv dilution tested, chip was regenerated with 1.5 μM glycine buffer (GE-Healthcare), run for 30 s at 10 μl.min^−1^. Affinities were calculated using the BIAevaluation software (GE-Healthcare) according to Langmuir adsorption model and results were verified by internal consistency tests [45].

### Computational analysis

The scFv sequences were analysed at the nucleotide level with IMGT/V-QUEST tool (http://www.imgt.org) to identify the closer human germline genes [46–48]. They were also analysed at the peptide level with IMGT/DomainGapAlign tool (http://www.imgt.org) to calculate the Germinality Index (GI). The GI corresponds to the percentage of identity at the peptide level between the framework regions of the isolated scFvs and those encoded by the most similar human germline sequences identified with IMGT/DomainGapAlign; here the GI is used as an indicator of scFv immunogenicity.

The G-score is used as a second indicator of the immunogenicity. G-score compares the selected scFv peptide sequences with human expressed antibodies present in the Kabat database belonging from the same family (i.e. derived from the same human germline gene) and is available online on University College London website (http://www.bioinf.org.uk/abs/) [29, 49].

### Hemidiaphragm assay

Left phrenic nerve-hemidiaphragm preparations were excised from male inbred mice (Balb/c) and installed in a 6 mL organ bath maintained at 37 °C containing Krebs-gelatin buffer gassed with 95 % O_2_–5 % CO_2_ bubbled through the buffer. Indirect stimulation *via* the phrenic nerve was continuously supplied with a supramaximal voltage (~3 V, 1 Hz, 0.2 ms) and the resulting muscle contractions were measured with an isometric force transducer (FMI Gmbh) linked to a ML110 bridge amplifier and a Powerlab / 4SP 4 channel recorder (ADInstruments). The hemidiaphragm resting tension was increased in a stepwise manner during the equilibration period, until reproducible twitches were observed. Once the muscle twitch response to nerve stimulation had stabilized and remained of a constant magnitude for at least 30 min without further adjustment, the Krebs buffer was replaced with 6 mL of toxin in Krebs solution and stimulation was resumed.

Toxin-induced paralysis was defined as a 50 % decrease in the muscle twitch response to neurogenic stimulation, based on the magnitude of the contractions just before the addition of the toxin. The myotoxic effects of the toxin were also assessed by applying a short burst of direct (muscle) stimulation (~30 V, 1 Hz, 1 ms,) before adding the toxin and at the end of the experiment.

Toxin neutralization by the scFv preparations was assessed by mixing 20 LD_50_.mL^−1^ (87 pg.mL^−1^) of BoNT/A_1_ holotoxin or 10 LD50.mL^−1^ of BoNT/A_2_ in complex form with concentrations of the scFv preparations ranging from 0.1 to 27 μg.mL^−1^ and by incubating for 30 min at 37 °C before applying the mixture to the tissue. The toxin dose considered optimal for antibody inhibition studies is typically that inducing about 80 % maximum paralytic activity on the dose-response curve (ensuring optimal precision), unless further scFv characterization requires weaker paralytic activity. After draining the organ baths, the toxin/scFv mixture was added to the bath and twitch responses were recorded for up to 295 min or until the twitch tension was no longer detectable. The time to 50 % paralysis after the addition of the mixture composed of BoNT and scFv was determined by fitting to the linear part of the paralysis curve [38, 50]. In the presence of a scFv, the paralysis is considered as significantly delayed, if the time to the 50 % paralysis is delayed by more than 10 min. The variability of 50 % paralysis time measurements using the BALB/c mice is minimal whatever the dose of BoNT/A with standard errors of the mean ranging from 1.6 min to 7.7 min at each dose level [50]. Curve fitting for calculation of 50 % paralysis times was performed using Graph Pad Prism. 5.0 software (Graphpad). The neutralization of toxin activity was proportional to the ability of the scFv to delay BoNT-induced paralysis. Greater antibody neutralizing potency was associated with the requirement of a longer period of time for the hemidiaphragm to reach 50 % paralysis for the same dose of toxin.

## Additional files

Additional file 1:
**Retroamplification of RNA coding the Fd fragment of the γ chain and κ light chain.** (PDF 1310 kb) Additional file 2:
**Count of eluted phage and evaluation of their specificity.** (PDF 16 kb) Additional file 3:
**Sensorgram of the scFv A1HC38 obtained in surface plasmon resonance.** (PDF 494 kb) Additional file 4:
**G-score of the 24 scFvs with affinity better than 10 nM.** (PDF 19 kb)

## Abbreviations

BoNT: Botulinum neurotoxin
CDC: Centers for Disease Control and Prevention
GI: Germinality index
HC: Heavy chain
LC: Light chain
LD_50_: 50 % lethal dose
scFv: Single chain fragment variable
SNAP25: Synaptosomal-associated protein of 25 kDa
SNARE: Soluble N-ethylmaleimide-sensitive factor attachment protein receptor
Fab: Fragment antigen binding
