# Supplementary material for: Isolation of nanomolar scFvs of non-human primate origin, cross-neutralizing botulinum neurotoxins A1 and A2 by targeting their heavy chain
Source: BMC Biotechnol. 2015 Sep 17;15:86. doi: 10.1186/s12896-015-0206-0 (PMC4574468; doi:10.1186/s12896-015-0206-0)
Supplement: Additional file 1: — Retroamplification of RNA coding the Fd fragment of the γ chain and κ light chain. (PDF 1310 kb) [file 12896_2015_206_MOESM1_ESM.pdf]

Additional file 1: Retroamplification of RNA coding the Fd fragment of the γ chain and κ light chain.

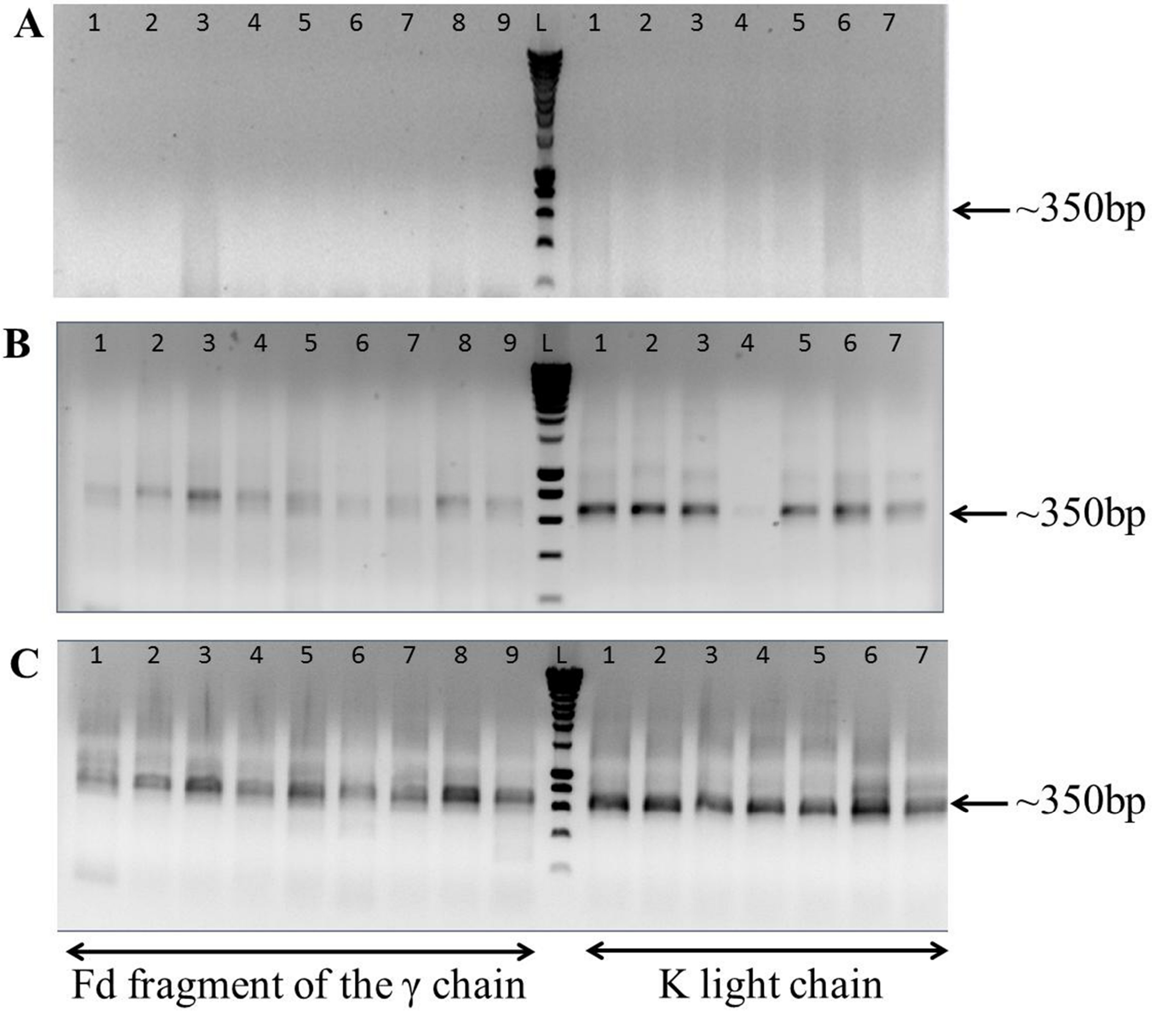

DNA coding the Fd fragments of the γ chain and κ light chains were amplified with 9and 7 specific primer sets, respectively. The corresponding products of amplification are composed of approximately 350 bp. 1-9: primer sets used for the amplification of the DNA coding the Fd fragments of the γ chain. L: Ladder. 1-7: primer sets used for the amplification of the DNA coding the κ light chains. **A**: PCR amplification of the DNA coding for the antibody variables domains, starting from bone marrow sampled just before the final immunization of the

macaque. **B**: Non-optimal PCR amplification of the DNA coding for the antibody variables domains, starting from the bone marrow sampled 3 days after the final immunization. **C**: optimal PCR amplification of the DNA coding for the antibody variables domains, starting from the bone marrow sampled 10 days after the final immunization. The amplification obtained at the 10th day was considered as optimal compared to the other days. Each band of the ladder (line L) corresponds to 100 pb.
