# Supplementary material for: Isolation of nanomolar scFvs of non-human primate origin, cross-neutralizing botulinum neurotoxins A1 and A2 by targeting their heavy chain
Source: BMC Biotechnol. 2015 Sep 17;15:86. doi: 10.1186/s12896-015-0206-0 (PMC4574468; doi:10.1186/s12896-015-0206-0)
Supplement: Additional file 2: — Count of eluted phage and evaluation of their specificity. (PDF 16 kb) [file 12896_2015_206_MOESM2_ESM.pdf]

Additional file 2: Count of eluted phage and evaluation of their specificity.

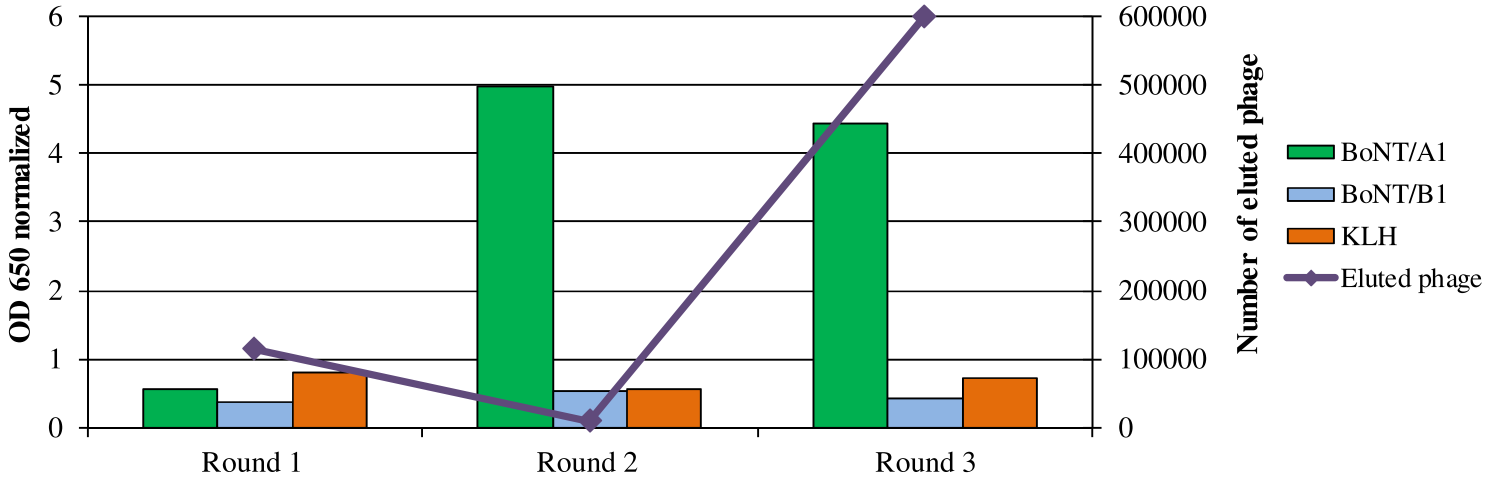

The number of phages (clones) eluted were counted after each round of panning, and their reactivity against BoNT/A_1, BoNT/B_1 or KLH was evaluated by phage-ELISA at the end of the panning. All antigens were coated at the concentration of 5 µg.mL^{-1}.
