# Supplementary material for: Isolation of nanomolar scFvs of non-human primate origin, cross-neutralizing botulinum neurotoxins A1 and A2 by targeting their heavy chain
Source: BMC Biotechnol. 2015 Sep 17;15:86. doi: 10.1186/s12896-015-0206-0 (PMC4574468; doi:10.1186/s12896-015-0206-0)
Supplement: Additional file 3: — Sensorgram of the scFv A1HC38 obtained in surface plasmon resonance. (PDF 494 kb) [file 12896_2015_206_MOESM3_ESM.pdf]

Additional file 3: Sensorgram of the scFv A1HC38 obtained in surface plasmon resonance.

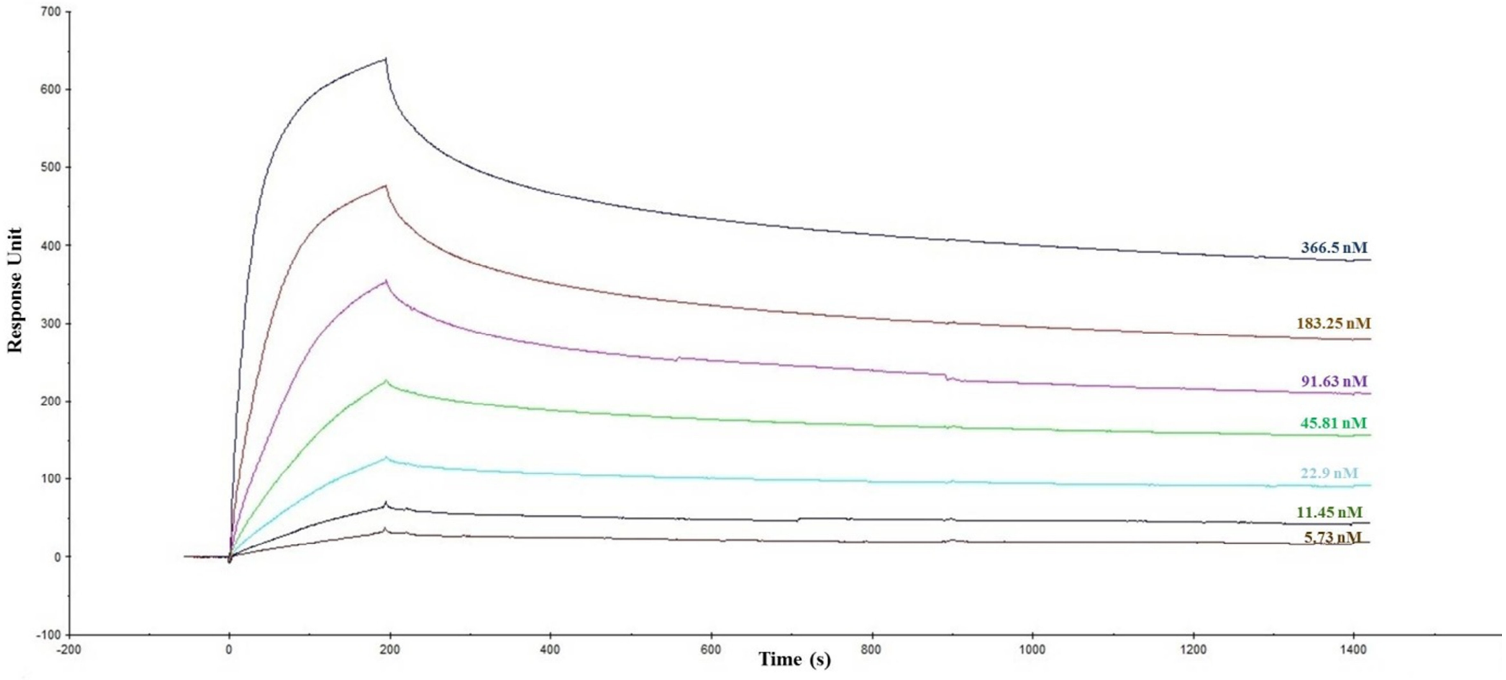

The affinity of the scFv A1HC38 was determined in surface plamon resonance (Biacore® technology). The affinity of the scFv A1HC38 was determined with injections of the scFv at concentrations ranging from 366.5 nM to 5.73 nM on a CM5-chip coated with BoNT/A_1. The intensity of the response was reported in the figure as "response unit". The affinity of the scFv A1HC38 was measured at 1.9 nM ; $K_{on} = 6.34x10^4 M^{-1}.S^{-1}$, $K_{off} = 1.2x10^{-4} S^{-1}$.
