# Supplementary material for: Isolation of nanomolar scFvs of non-human primate origin, cross-neutralizing botulinum neurotoxins A1 and A2 by targeting their heavy chain
Source: BMC Biotechnol. 2015 Sep 17;15:86. doi: 10.1186/s12896-015-0206-0 (PMC4574468; doi:10.1186/s12896-015-0206-0)
Supplement: Additional file 4: — G-score of the 24 scFvs with affinity better than 10 nM. (PDF 19 kb) [file 12896_2015_206_MOESM4_ESM.pdf]

Additional file 4: G-score of the 24 scFvs with affinity better than 10 nM.

	G-Score		
Clone name	**Heavy chain**	**Light chain**	**Mean G-score**
A1HC81	-2.51	0.491	-1.01
A1HC62	-1.72	-0.34	-1.03
A1HC67	-0.70	-1.43	-1.06
A1HC58	-1.91	-0.35	-1.13
A1HC74	-0.83	-1.56	-1.19
A1HC65	-2.64	0.13	-1.25
A1HC43	-0.93	-1.60	-1.27
**A1HC45**	**-3.01**	**0.36**	**-1.32**
A1HC34	-2.93	0.04	-1.44
A1HC49	-2.83	-0.09	-1.46
A1HC26	-2.81	-0.11	-1.46
**A1HC17**	**-1.73**	**-1.32**	**-1.53**
A1HC32	-2.11	-1	-1.55
A1HC33	-2.51	-0.58	-1.55
A1HC64	-2.64	-0.55	-1.6
A1HC3	-2.72	-0.66	-1.69
**A1HC38**	**-2.12**	**-1.38**	**-1.75**
A1HC7	-2.64	-0.86	-1.75
A1HC68	-2.64	-0.91	-1.77
A1HC31	-2.82	-0.79	-1.8
A1HC39	-2.95	-0.7	-1.83
A1HC80	-2.74	-0.95	-1.84
A1HC6	-1.15	-2.95	-2.05
A1HC47	-2.73	-2.01	-2.37

The G-score was calculated for each of the 24 selected scFv (VH, VL, mean G-Score) using UCL web server tool. The scFvs were ranked according to their predictive tolerance.
